# Assessment of iron overload in a cohort of Sri Lankan patients with transfusion dependent beta thalassaemia and its correlation with pathogenic variants in *HBB, HFE, SLC40A1, and TFR2* genes

**DOI:** 10.1186/s12887-022-03191-8

**Published:** 2022-06-15

**Authors:** Ruwangi Dissanayake, Nayana Samarasinghe, Samantha Waidyanatha, Sajeewani Pathirana, Nilaksha Neththikumara, Vajira H. W. Dissanayake, Kalum Wetthasinghe, Lallindra Gooneratne, Pujitha Wickramasinghe

**Affiliations:** 1 Department of Paediatrics, Faculty of Medicine, University of Colombo, Colombo, Sri Lanka; 2 Lady Ridgeway Hospital for Children, Colombo, Sri Lanka; 3 Human Genetics Unit, Faculty of Medicine, University of Colombo, Colombo, Sri Lanka; 4 Department of Pathology, Faculty of Medicine, University of Colombo, Colombo, Sri Lanka

**Keywords:** Transfusion dependent beta thalassaemia, T2* MRI, Iron overload

## Abstract

**Background:**

Iron overload (IO) is a complication in transfusion dependent beta thalassaemia (TDT). Pathogenic variants in genes involving iron metabolism may confer increased risk of IO. The objective of this study was to determine the magnitude of the cardiac and hepatic IO and determine whether pathogenic variants in *HFE, SLC40A1* and *TFR2* genes increase the risk of IO in a cohort of TDT patients in Sri Lanka.

**Materials and Methods:**

Fifty-seven (57) patients with TDT were recruited for this study**.** Serum ferritin was done once in 3 months for a period of one year in all. Those who were ≥ 8 years of age (40 patients) underwent T2* MRI of the liver and heart. Fifty-two (52) patients underwent next generation sequencing (NGS) to identify pathogenic variants in *HBB, HFE, SLC40A1* and *TFR2* genes.

**Results:**

The median age of the patients of this cohort was 10 years. It comprised of 30 (52.6%) boys and 27 (47.4%) girls. The median level of serum ferritin was 2452 ng/dl. Hepatic IO was seen in 37 (92.5%) patients and cardiac IO was seen in 17 (42.5%) patients. There was no statistically significant correlation between serum ferritin and hepatic or cardiac IO.

Thirty-two (61.5%), 18 (34.6%), 2 (3.8%) of patients were homozygotes, compound heterozygotes and heterozygotes for pathogenic variants in the *HBB* gene. Eight (15.4%) and 1 (1.9%) patients were heterozygotes for pathogenic and likely pathogenic variants of *HFE* genes respectively. There were no pathogenic variants for the TfR2 and *SLC40A1* genes. The heterozygotes of the pathogenic variants of the *HFE* were not at increased risk of IO.

**Conclusions:**

Cardiac T2* MRI helps to detect cardiac IO in asymptomatic patients. It is important to perform hepatic and cardiac T2* MRI to detect IO in patients with TDT. There was no statistically significant correlation between pathogenic variants of *HBB* and *HFE* genes with hepatic and cardiac IO in this cohort of patients.

## Introduction

Transfusion dependent β thalassaemia (TDT) is a single gene disorder characterised by a quantitative deficiency of beta globin chains. Almost 300 pathogenic variants causing beta thalassaemia have now been described [1]. Patients with TDT require regular blood transfusion to survive, and without adequate transfusion support, they would suffer several complications and a short life span [2].

Regular blood transfusions as well as increased intestinal iron absorption contributes to IO in patients with TDT leading to morbidity and mortality. The human body lacks a physiological mechanism to remove excess iron resulting from blood transfusions. Each unit of packed red blood cells (RBC) contains 200–250 mg of elemental iron. The transfused RBCs are phagocytosed by the reticuloendothelial macrophages and the labile cellular iron is released into the plasma to bind transferrin. After transferrin is saturated, the non-transferrin bound iron is transported through calcium channels to the liver and heart.

Cardiac IO is an important cause of mortality in these patients. Hepatic IO results in liver fibrosis, cirrhosis and hepatitis [3].

Secondary genetic modifiers modulate the complications of beta thalassaemia. Several genes that affect iron homeostasis have now been characterized and they include *HFE*, transferrin receptor 2 (*TfR2*), and ferroportin (*SLC40A1*) genes. Pathogenic variants in genes responsible for iron homeostasis can aggravate the complications of IO and accelerate the development of organ specific haemosiderosis in patients with TDT [4]. The haemochromatosis gene *HFE* encodes the major histocompatibility complex (MHC) class-1like protein HFE that binds beta-2 microglobulin. HFE influences iron absorption by modulating the expression of hepcidin, the main controller of iron metabolism [5]. *TfR2* is postulated to be part of a signaling system that modulates hepcidin expression. The molecular details of *TfR2*s role in this system is unclear. *TfR2* is predicted to bind the iron carrier transferrin when the iron saturation of transferrin is high [6]. *SLC40A1* gene encodes a main iron export protein-ferroportin 1 [7]. Pathogenic variants of *SLC40A1* cause physicochemical changes in this protein and cause iron overload by an intracellular retention mechanism [8].

Mutations leading to ferroportin that is either not present at the cell surface or has defective iron export activity are associated with iron deposition in kupfer cells and low transferrin saturation. Mutated ferroportin that cannot be internalized after hepcidin binding gain of function are associated with iron deposition in hepatocytes and elevated transferrin saturation [9 10]. Pathogenic variants in the *HFE,TFR2 and SLC40A1* genes are seen in hereditary haemochromatosis (HH). IO in TDT is also seen with these pathogenic variants.

*HFE* C282Y and H63D pathogenic variants are common causes of IO in different populations [4]. In a cohort of Iranian patients with beta thalassaemia major it was shown that the *HFE* H63D pathogenic variant was a significant determinant of elevated serum ferritin [4]. Abnormal Ferroportin has been identified to cause HH. Pathogenic variants in the Ferroportin gene (*SLC40A1*) results in Haemochromatosis type 4. Co inheritance of pathogenic variants in the *HBB* gene with pathogenic variants in the *SLC40A1* gene may aggravate the IO and require more aggressive therapy [11]. Pathogenic variants in the *TfR2* gene are seen in Haemochromatosis type 3 [12].

IO is monitored by regular serum ferritin levels. Though testing for ferritin level is widely available it has the disadvantage of being an acute phase reactant. Liver biopsy with estimation of liver iron content is the gold standard for monitoring hepatic IO, however it has the disadvantage of being invasive. The T2* MRI is now recommended for measuring hepatic and cardiac IO. It does not expose the patient to radiation, contrast or sedation.

Our centre at the Lady Ridgeway Hospital for Children in Colombo, Sri Lanka was the first centre to provide T2* MRI to assess hepatic and cardiac iron status for patients with TDT in Sri Lanka. The cohort of patients described in this study is the first set of patients who underwent this assessment in Sri Lanka. The objective of this study was to determine the magnitude of the cardiac and hepatic IO and determine whether pathogenic variants in *HFE, SLC40A1* and *TFR2* genes increase the risk of IO in this cohort of TDT patients.

## Material and methods

### Study Population

The study population consisted of fifty-seven (57) patients with TDT. Patients with **β t**halassaemia based on HPLC and/or genetic testing on regular transfusions (3–4 weekly) as decided by a consultant paediatrician were recruited from the paediatric wards of the Lady Ridgeway Hospital for Children, Colombo, Sri Lanka after obtaining informed written consent from their parents or guardians according to a protocol approved by the Ethics Review Committee of the Faculty of Medicine, University of Colombo. The patients were recruited when admitted for blood transfusion. The study period was one year. At the time of recruitment the patients were examined for evidence of hepatomegaly, splenomegaly and cardiomegaly. Fifty patients (87.7%) were on regular (3–4 weekly) blood transfusions for ≥ 2 years at the time of recruitment for the study and seven (12.3%) patients were on regular blood transfusions for less than 1 year at the time of recruitment.

In addition to the regular blood transfusions the patients were on iron chelation which was started once the serum ferritin was > 1000 ng/ml [13]. Eighteen (31.6%) patients were only on Deferasirox (DFX) given orally. Three (5.3%) were only on Desferioxamine (DFO) given subcutaneous (SC). Sixteen (28.1%) were on DFX and DFO given SC. Nine (15.8%) were on DFX and DFO given intravenously (IV). Eleven (19.3%) were on DFX and DFO given SC and IV. The preferred chelator was Deferasirox as it can be given orally and is given only once a day. However those with a serum ferritin of > 2000 ng/ml received combined Deferasirox and Desferioxamine given subcutaneously. Those with cardiac iron overload were offered Deferasirox, Desferioxamine given subcutaneously and intravenously.

### Investigations

Their pre-transfusion haemoglobin (Hb) was recorded prior to each transfusion over a period of one year. The post transfusion Hb and serum ferritin was recorded once in 3 months during the same period.

Forty (40) patients ≥ 8 years of age underwent T2* MRI to look for evidence of hepatic and cardiac IO at the department of radiology of the hospital.

### Genotyping

Peripheral venous blood of the patients was collected into the Ethylenediamine tetraacetic acid (EDTA) tubes at the time of inserting cannula for blood transfusion. DNA was extracted from the white blood cells from the samples by using the QIAamp DNA Mini Kit according to the manufacturer’s protocol. A custom made targeted resequencing panel for haematological disorders, designed in-house and manufactured by Illumina, was used to enrich the extracted DNA. Susceptible genes and gene targets were identified by examining already published scientific literature. The enriched libraries were sequenced on an Illumina® MiSeq Next generation sequencer.

### Bioinformatics analysis

High quality paired end FASTQ files obtained from the Miseq platform were aligned to the reference human genome GRCh37 primary assembly by Burrows Wheel Aligner (BWA) using the BWA-MEM algorithm with default parameters. Indexing was performed by sequence alignment map (SAM) tools and deduping of reads were performed using the Genome Analysis Tool Kit (GATK) followed by realigning of the deduped binary alignment map (BAM) file.

Annotation of the resulted variant call format (VCF) file was done using SNP-eff with clinical databases; ClinVar, OMIM and population frequency databases; 1000 Genomes, HapMap and Exome aggregation Consortium (ExAC). In silico tools such as MutationTaster, SIFT, PolyPhen2, and Provean were used for functional prediction. GeneSplicer was used for splice site detection.

Secondary analysis was performed using an in-house bioinformatics pipeline at the Human Genetics Unit, specifically developed and validated for NGS single nucleotide variant analysis. Variant position along with the other details such as clinical significance, population frequency and insilico prediction was obtained as a text file in tab separated format (tsv) as the final output of the pipeline. The genetic variants obtained from the annotation were further scrutinized manually, considering their availability in public databases, their conservation, and their functional impact on the protein to identify the disease-causing variants.

Secondary variant analysis was specifically performed to identify clinically significant variants in the *HBB, HFE, TFR2, SLC40A1* (*Ferroportin*) genes. A locus specific database named, HbVar, was used to correlate the genotype and phenotype data for human Hemoglobin Variants and Thalassemia Mutations.

### MRI

A Siemens 1.5 T MRI scanner was used with an echo time (TE) of 1.3 ms and a response time of 200 ms. The slice thickness used in the MRI T2* was 10 mm with matrix size 2.7 × 10 mm. Fat suppression was not used. Multiple TEs were obtained in single breath hold for both liver iron concentration (LIC) and myocardial iron concentration (MIC). Decay curves were generated by a universal exponential decay formula. LIC calculation was based on Hankins et al. 2009 and MRI T2* was converted to reciprocal R2* by the mathematical formula R2* (Hz) = 1000/T2 (ms), and truncation model was applied [14]. T2* axial images were obtained for LIC. Mid ventricular short axis view was taken for MIC with electrocardiogramme gating. MIC calculation was based on Carpenter JP et al. 2011 [15]. Multiple TEs were taken from the inter ventricular septum. All the studies were done by one MRI technician without general anaesthesia or any mode of sedation. Free breathing technique was used in some patients who couldn’t cooperate adequately with breath holding.

The range of hepatic and cardiac iron overload is as given.

**Table:** 

Hepatic		
Normal - T2*>11.4ms	R2*<88Hz	LIC<2.0mg/g
Mild IO - T2*3.8-11.4ms	R2*88-263Hz	LIC 2-7mg/g
Moderate IO - T2*1.8-3.8ms	R2*263-555Hz	LIC 7-15mg/g
Severe IO - T2*<3.8ms	R2*>555Hz	LIC>15mg/g
Cardiac		
Normal - T2*>20ms	R2*<50Hz	MIC<1.16mg/g
Mild IO - T2*15-20ms	R2*50-66.5Hz	MIC 1.16-1.65mg/g
Moderate IO-T2*10-15ms	R2*66.6-100Hz	MIC 1.65-2.71mg/g
Severe IO - T2*<10ms	R2*>100Hz	MIC>2.71mg/g.

## Results

There were 57 patients.

The median age of this cohort was 10 years. There were 30 (52.6%) males and 27 (47.4%) females. Hepatomegaly was seen in twenty-seven (47.3%) and splenomegaly in sixteen (28%) of patients. Seven (12.3%) had undergone splenectomy. None of the patients had cardiomegaly.

The median pre transfusion Hb was 9.0 g/dl and the median post transfusion Hb was 13.3 g/dl. The median of the total volume of blood transfusion per year was 255 ml/kg. They had a median serum ferritin of 2452 ng/dl. The median age of the 40 patients who underwent hepatic and cardiac T2* MRI was 11.6 years. There were 24 (60%) males and 16 (40%) females.

The demographics and investigation findings of the patients according to hepatic and cardiac IO status is in Table 1.

**Table 1:** Characteristics of children with TDT according to hepatic and cardiac IO on T2*MRI

	Hepatic T2* MRI				Cardiac T2* MRI			
	Normal	Mild IO	Moderate IO	Severe IO	Normal	Mild IO	Moderate IO	Severe IO
Number (%)	3 (7.5%)	27 (67.5%)	10 (25%)	-	23 (57.5%)	7 (17.5%)	6 (15%)	4 (10%)
Median Age (years)	8.6	12.75	11.5	-	10.5	12.75	14.6	13.6
Median ferritin (ng/ml)	925	3411	3427	-	2389	5366	3168	4839
Median Pre Transfusion Hb (g/dl)	8.7	9.0	9.4	-	9.0	8.9	9.2	8.8
Median Post transfusion Hb (g/dl)	13.1	13.0	14.1	-	13.4	13.6	12.7	12

Correlation of hepatic and cardiac MRI with age and serum ferritin is shown in Table 2. Pearson correlation coefficient test revealed no statistically significant correlation between cardiac T2* or MIC with age and serum ferritin. Similarly there was no statistically significant correlation between hepatic T2* or LIC with age and serum ferritin.

**Table 2:** Correlation of hepatic and cardiac MRI with age and serum ferritin

	Cardiac T2*	MIC	Hepatic T2*	LIC
Age	*r* = -0.078	*r* = 0.215	*r* = -0.190	*r* = 0.054
	*p* = 0.634	*p* = 0.183	p = 0.241	*p* = 0.739
Ferritin	*r* = -0.289	*r* = -0.225	*r* = -.191	*r* = 0.012
	*p* = 0.071	*p* = 0.163	*p* = 0.237	*p* = 0.943

There was a positive correlation between cardiac T2* and hepatic T2* and between MIC and LIC (*p* = 0.331, *r* = 0.158). Both were not statistically significant.

Out of the 52 patients who underwent next generation sequencing, the pathogenic variants in the *HBB* gene (Nomenclature with respect to NM_000518.5) were, c.92+5G>C (IVS1-5G>C), c.92+1G>A(IVS1-1G>A), c.315+1G>A(IVS2-1G>A), NM_000518.4(HBB):c.92G>C (p.Arg31Thr), c.47G>A (p.Trp16Ter), c.93-1G>C (IVS I-130G>C),c.51del, c.2T>C ,c.91A>C, c.126del, c126_129delCTTT, and c.46delT. Thirty two (61.5%) were homozygotes, eighteen (34.6%) were compound heterozygotes and two (3.9%) were heterozygotes with unusual severity. 

The pathogenic and likely pathogenic variants found in the *HFE* gene were respectively, NM_000410(*HFE*):c.187C > G (H63D) and c.262A > T. Eight (15.4%) were heterozygote for the c.187C > G variant and one (1.9%) was heterozygote for the c.262A > T variant. C.262A > T was a novel variant, which has not been mentioned in population frequency databases or scientific literature before. It was classified as a likely pathogenic variant. The NM_000410.3(HFE):c.845G > A (p.Cys282Tyr) variant was not seen in this cohort. There were no pathogenic variants in the *TfR2* and *SLC40A1* gene in this cohort.

Out of the thirty-two (32) patients who were homozygous for the *HBB* pathogenic variants, 25 underwent cardiac and hepatic MRI. The results of these are given in Table 3. Twelve (12) patients who were compound heterozygous for the *HBB* gene underwent hepatic and cardiac MRI. There was no statistically significant difference between the means of serum ferritin, cardiac T2*, MIC, hepatic T2* and LIC between these 2 groups on independent sample t test.

**Table 3:** IO with pathogenic variants in *HBB*, *HFE*, *TFR2*, and *SLC40A1* (*Ferroportin*) genes

Gene		*HBB*	*HFE*	*SLC40A1*	*TFR2*
			(H63D)		
Homozygotes	Number	32	0	0	0
	Median serum ferritin (ng/ml)	2546	-	-	-
	Median Hepatic T2* (ms)	4.1	-	-	-
	Median LIC (mg/g)	6.45	-	-	-
	Median Cardiac T2* (ms)	20.6	-	-	-
	Median MIC (mg/g)	1.12	-	-	-
Compound	Number	18	0	0	0
Heterozygotes	Meadian serum ferritin (ng/ml)	2490	-	-	-
	Median Hepatic T2* (ms)	4.8	-	-	-
	Median LIC (mg/g)	5.4	-	-	-
	Median Cardiac T2* (ms)	27.15	-	-	-
	Median MIC (mg/g)	0.82	-	-	-
Heterozygotes	Number	2	8	0	0
	Median serum ferritin (ng/ml)	-	1791.5	-	-
	Median Hepatic T2* (ms)	-	4.6	-	-
	Median LIC (mg/g)	-	5.72	-	-
	Cardiac T2* (ms)	-	14.8	-	-
	Median MIC (mg/g)	-	1.76	-	-

Forty-three (43) patients who had no pathogenic variants in the *HFE* gene had a mean serum ferritin of 3659 (± 2832) ng/ml. The eight (8) patients who were heterozygous for the H63D pathogenic variant in the *HFE* gene had a mean serum ferritin of 2574 (± 2183) ng/ml. Independent samples t test showed no statistically significant difference between the mean serum ferritin of these 2 groups. Four patients, who were heterozygotes for the H63D pathogenic variant of the *HFE* gene and 33 patients who had no pathogenic variants for the *HFE* gene underwent cardiac and hepatic MRI T2*. The mean cardiac T2* and MIC in the patients with the pathogenic variants of the *HFE* were 19.7 (± 14.7) and 1.86 (± 1.25). In the group with no pathogenic variants of the *HFE* gene had a mean cardiac T2* of 24.25 (± 11.14) and mean MIC of 1.38 (± 1.17). Independent samples t test showed no statistically significant difference of the mean cardiac T2* or MIC between these 2 groups. The mean hepatic T2* was 4.76 (± 1.64) in the *HFE* heterozygote group and 5.77 (± 4.0) in the group with no pathogenic variants in the *HFE* gene with no statistically significant difference on independent sample t test. The mean LIC was 5.99 (± 2.24) in the *HFE* heterozygotes and 5.97 (± 3.06) in those with no pathogenic variants of the *HFE* gene. There was no statistically significant difference between the mean LIC of these 2 groups.

## Discussion

This cohort of TDT patients was followed up according to the Thalassaemia International Federation (TIF) guideline [7]. Accordingly the MRI T2* of the liver and heart was done in those ≥ 8 years of age. Cardiac IO was seen as early as the first decade of life. The youngest being 8.6 years of age. Hepatic IO was also seen in the first decade of life. The youngest was 8.7 years.

In a study done in Italy, patients as young as 6 years of age showed evidence of cardiac IO on MRI [16]. However, John C Wood et al.in their study among 77 patients with thalassaemia major from Italy and Los Angeles found that there was no cardiac IO in patients less than 9.5 years of age [17]. Berdoukas et al.in a study done in Los Angeles, described hepatic IO in patients with thalassaemia major less than 3.5 years of age. In the same study 10% of patients had cardiac IO during the first 10 years of life [18].

In our study hepatic IO was commoner than cardiac IO with 92.5% of patients having hepatic IO and 42.5% having cardiac IO. Similar results were shown in studies done in other regions of the world. In a study done in Thailand to assess IO in patients with thalassaemia major, 87.1% had hepatic IO and 18.6% had cardiac IO [19]. Among Egyptian patients with TDT, 75% had IO in the liver and 8.7% had evidence of IO in the heart [20]. A study done in Iran showed hepatic IO in 41.6% and cardiac IO in 38.3% of TDT patients [4].

There was no statistically significant correlation between cardiac MRI T2* and MIC nor serum ferritin. The correlation between hepatic MRI T2*, LIC and serum ferritin was not statistically significant. There was no statistically significant correlation between hepatic IO and cardiac IO. In a study done in Thailand there was no significant correlation between cardiac T2* and serum ferritin. In this study there was a negative correlation between liver iron concentration and serum ferritin [19]. Kiran Suthar et al.in their study done on a cohort of Indian children with TDT concluded that serum ferritin was a poor indicator of predicting IO in the liver and heart [21]. Several other studies have shown dissimilar correlation strengths ranging from no correlation to low correlation between serum ferritin and cardiac iron content [22]. Therefore serum ferritin is a poor indicator of cardiac or hepatic IO and it is important to perform MRI for detection of hepatic and cardiac IO.

It is important to perform T2* MRI in patients with TDT to detect cardiac IO prior to becoming symptomatic. Once cardiac dysfunction is detected by echocardiogramme survival is reduced in these patients suggesting a late stage of detection of disease by this assessment [23]. Alessia Pepe et al.in their study done to look at cardiac complications in patients with TDT in Italy concluded that pre- clinical iron deposition is best assessed by cardiac T2* MRI and serum ferritin and liver iron concentration are not good predictors of cardiac complications [24].

The three most common pathogenic variants of the *HFE* gene causing IO are p.C282Y (exon 4; c.845G → A; rs1800562); p.H63D (exon 2; c.187C → G; rs1799945); and p.S65C (exon 2; c.193A → T; rs1800730) [5]. Amit Kumar Tiwari et al. in their study among 50 patients with TDT in India detected only the H63D pathogenic variant among 8 patients. The C282Y and S65C pathogenic variants were not seen [25]. Similarly there were no patients with C282Y pathogenic variants in the study conducted in India by Agarwal et al. among patients with thalassaemia syndrome. Sixteen percent of patients with thalassaemia syndrome had H63D pathogenic variant in this study (3 homozygotes and 43 heterozygotes) [26]. The C282Y pathogenic variant is relatively rare in the Indian subcontinent as compared to the rest of the world where it is relatively more common. Agarawal et al. described an increase in serum ferritin in the thalassaemia syndrome patients who had the H63D pathogenic variant [26].

Aysen Turedi et al. in their study among 33 patients with beta thalassaemia major found only H63D pathogenic variant among 6 patients (5 heterozygotes and 1 homozygote) [27]. Similarly, in our study 15.4% had H63D pathogenic variant and the C282Y and S65C pathogenic variants were not seen.

Previous studies have shown higher serum ferritin in TDT patients with these pathogenic variants of *HFE*. However, they were unable to show a statistically significant increase in IO on hepatic and cardiac T2* MRI. In a study done in Iran among 60 patients with TDT C282Y pathogenic variant was not seen. The H63D pathogenic variant was detected among 20% of the study population. There was a significant increase in serum ferritin among these carriers but no increase in cardiac or hepatic haemosiderosis on T2* MRI [4]. Azza Aboul Enein et al.in their study among 50 Egyptians patients with TDT described 10% H63D heterozygotes. The C282Y and S65C pathogenic variants were not seen. There were higher levels of serum ferritin among the H63D carriers [28].

Romina Rahmani et al. described a significant rise in serum ferritin among TDT patients H63D and C282Y pathogenic variants [29]. Zekavat OR et al. described a significant rise in serum ferritin without an increase in hepatic and cardiac IO in a study among TDT patients with H63D pathogenic variant. In their study among 253 patients with TDT 35.9% had H63D pathogenic variant. The C282Y pathogenic variant was present only in one patient [30].

Different types of pathogenic variants in ferroportin were described causing type 4 haemochromatosis. Montosi et al. determined linkage of autosomal dominant haemochromatosis to 2q32 and demonstrated a missense mutation in the ferroportin gene (*SLC40A1*): GCC to GAC change resulting an A77D substituition [31].

Inactivating mutations in TfR2 cause Haemochromatosis type 3 [6]. In Central southern Italy pathogenic variants of *TFR2* including novel variants were described in patients with HH. In our cohort there were no pathogenic variants of *TFR2 *[32].

## Conclusions

Hepatic and cardiac IO was seen in this cohort of TDT patients. The onset was as early as the first decade of life. The cardiac T2* MRI helps to detect IO in asymptomatic patients and will help optimise iron chelation and prevent mortality. Therefore it is important to perform T2* MRI to look for evidence of IO in asymptomatic patients. The small sample size was a limitation in this study. Therefore studies involving a larger number of patients need to be done to establish a correlation with IO and pathogenic variants in *HFE*, *SLC40A1* and *TFR2* genes.

## Data Availability

Data generated in this study is not available for ethical/patient confidentiality reasons.

## Abbreviations

IO: Iron overload
TDT: Transfusion dependent beta thalassaemia
NGS: Next generation sequencing
RBC: Red blood cells
MHC: Major histocompatibility complex
HH: Hereditary haemochromatosis
DFX: Deferasirox
DFO: Desferioxamine
SC: Subcutaneous
IV: Intravenously
Hb: Haemoglobin
EDTA: Ethylenediamine tetraacetic acid
BWA: Burrows Wheel Aligner
SAM: Sequence alignment map
GATK: Genome Analysis Tool Kit
VCF: Variant call format
ExAC: Exome aggregation Consortium
TSV: Tab separated format
TE: Echo time
LIC: Liver iron concentration
MIC: Myocardial iron concentration
TIF: Thalassaemia International Federation

## References

[1] Thein SL (2013). The molecular basis of β-thalassemia. Cold Spring Harb Perspect Med..

[2] Viprakasit V, Origa R, Cappelini MD, Cohen A, Porter J, Taher A, Viprakasit V (2014). Genetic basis, pathophysiology and diagnosis. Guidelines for the management of transfusion dependent thalassaemia (TDT).

[3] Taher AT, Saliba AN (2017). Iron overload in thalassemia: different organs at different rates. Hematology Am Soc Hematol Edu Program.

[4] Soltanpour MS, Davari K (2018). The correlation of cardiac and hepatic hemosiderosis as measured by T2* MRI technique with ferritin levels and hemochromatosis gene mutations in Iranian patients with beta thalassaemia major. Oman Med J.

[5] Barton JC, Edwards CQ, Acton RT (2015). HFE gene: Structure, function, mutations and associated iron abnormalities. Gene.

[6] Kleven MD, Jue S, Enns CA (2018). Transferrin Receptors TfR1 and TfR2 Bind Transferrin through Differing Mechanisms. Biochemistry.

[7] Pietrangelo A (2004). The ferroportin disease. Blood Cells Mol Dis.

[8] Brissot P, Loreal O (2016). Iron metabolism and related genetic diseases: a cleared land, keeping mysteries. J Hepatol.

[9] Lee PL, Gelbart T, West C, Barton JC (2007). SLC40A1c 1402G->A results in aberrant splicing, ferroportin truncation after glycine 330 and an autosomal dominant hemochromatosis phenotype. Acta Haematol-Basel.

[10] Zhang W, Lv T, Huang J, Ou X (2017). Type 4B hereditary hemochromatosis associated with a novel mutation in the SLC40A1 gene A case report and a review of the literature. Medicine (Baltimore).

[11] Agarwal S, Sankar VH, Tewari D, Pradhan M (2006). Ferroportin (SLC40A1) gene in thalassaemic patients of Indian descent. Clin Genet.

[12] Roetto A, Totaro A, Alberto Pipperno A, Pippa A, Longo F, Garozzo G (2001). New mutations inactivating transferrin receptor 2 in hemochromatosis type 2. Blood.

[13] Cappellini MD, Cohen A, Porter J, Taher A, Viprakasit V editors. Guidelines for the management of transfusion dependent thalassaemia (TDT) 3^rd^ ed. Nicosia (Cyprus): Thalassaemia International federation; 2014. 25610943

[14] Hankins JS, McCarville MB, Loeffler RB, Smeltzer MP, Onciu M, Hoffer FA (2009). R2* magnetic resonance imaging of the liver in patients with iron overload. Blood.

[15] Carpenter JP, He T, Kirk P, Roughton M, Anderson LJ, de Noronha SV (2011). On T2* magnetic resonance and cardiac iron. Circulation.

[16] Borgna- Pignati C, Meloni A, Guerrini G, Gulino L, Filosa A, Ruffo GB (2014). Myocardial iron overload in thalassaemia major How early to check?. Br J of Haematol.

[17] Wood JC, Origa R, Agus A, Matta G, Coates DT, Gallanello R (2008). Onset of cardiac iron loading in pediatric patients with thalassemia major. Haematologica.

[18] Berdoukas V, Nord A, Carson S, Puliyel M, Hofstra T, Wood J (2013). Tissue iron evaluation in chronically transfused children shows significant levels of iron loading at a very young age. Am J Hematol.

[19] Chaosuwannakit N, Makarawate P (2019). The value of magnetic resonance imaging in evaluation of myocardial and liver iron overload in a thalassaemia endemic population : a report from Northeastern Thailand. Pol J Radiol.

[20] Elfawal SK, Emaara DM, Shehata AA (2018). Assessment of hepatic and cardiac iron overload in thalassemia patients by magnetic resonance imaging : Our experience in Alexandria university. Egypt J radiol nucl med.

[21] Suthar K, Goyal VK, Sharma P, Deopa B, Rathore PS, Bishnoi RK (2018). Relationship between T2* magnetic resonance imaging-derived liver and heart iron content and serum ferritin levels in transfusion dependent thalassemic children. Asian J Transfus sci.

[22] Kirk P, Roughton M, Porter JB, Walker JM, Tanner MA, Patel J (2009). Cardiac T2* magnetic resonance for prediction of cardiac complications in thalassemia major. Circulation.

[23] Koonrungsesomboon N, Chattipacorn SC, Fucharoen S, Chattipakorn N (2013). Early detection of cardiac involvement in thalassemia : from bench to bedside perspective. World J of Cardiol.

[24] Pepe A, Meloni A, Gluseppe Rossi G, Midiri M, Missere M, Valeri G (2018). Prediction of cardiac complications of thalassemia major in the widespread cardiac magnetic resonance era: a prospective multicenter study by a multi parametric approach. Eur Heart J Cardiovasc Imaging.

[25] Tiwari AK, Behera TR, Kujur M, Singh AB (2016). Association of HFE gene mutation in thalassemia major patients. J Evid Based Med.

[26] Agarwal S, Tewari D, Arya V, Moorchung N, Tripathi R, Chaudhuri G (2007). Status of HFE mutation in thalassemia syndromes in north India. Ann Hematol.

[27] Turedi A, Oymak Y, Meşe T, Yaman Y, Bayraktaroglu S, Alpman A (2013). The effect of HFE polymorphisms on cardiac iron overload in patients with beta-thalassemia major. Pediatr Hematol Oncol.

[28] Enein AA, El Dessouky NA, Mohamed KS, Botros SK, El Gawad MF, Hamdy M (2016). Frequency of Hereditary Hemochromatosis (HFE) Gene Mutations in Egyptian Beta Thalassemia Patients and its Relation to Iron Overload. Maced J Med Sci.

[29] Rahmani R, Naseri P, Safaroghli-Azar A, Tarighi S, Hosseini T, Hojjati MT (2019). Investigation of correlation between H63D and C282Y mutations in HFE gene and serum ferritin level in beta-thalassemia major patients. Transfus Clin Biol.

[30] Zekavat OR, Zareian JM, Haghpanah S, Kargar JZ, Cohan N (2021). Association of HFE gene mutations with serum ferritin level and heart and liver iron overload in patients with transfusion-dependent beta-thalassemia. J Pediatr Hematol Oncol.

[31] Montosi G, Donovan A, Totaro A, Garuti C, Pignatti E, Cassanelli S (2001). Autosomal-dominant hemochromatosis is associated with a mutation in the ferroportin (SLC11A3) gene. J Clin Invest.

[32] Radio FC, Majore S, Binni F, Valiante M, Ricerca BM, De Bernardo C (2014). TFR2-related hereditary hemochromatosis as a frequent cause of primary iron overload in patients from Central-Southern Italy. Blood Cells Mol Dis.

